# Central Neuropathic Pain Development Modulation Using Coffee Extract Major Polyphenolic Compounds in Spinal-Cord-Injured Female Mice

**DOI:** 10.3390/biology11111617

**Published:** 2022-11-04

**Authors:** Roger Soler-Martínez, Meritxell Deulofeu, Anna Bagó-Mas, Petr Dubový, Enrique Verdú, Núria Fiol, Pere Boadas-Vaello

**Affiliations:** 1 Research Group of Clinical Anatomy, Embryology and Neuroscience (NEOMA), Department of Medical Sciences, University of Girona, E-17003 Girona, Catalonia, Spain; 2 Department of Anatomy, Division of Neuroanatomy, Faculty of Medicine, Masaryk University, 625 00 Brno, Czech Republic; 3 Department of Chemical Engineering, Agriculture and Food Technology, Polytechnic School, University of Girona, E-17003 Girona, Catalonia, Spain

**Keywords:** neuropathic pain, spinal cord injury, polyphenols, coffee extract, chlorogenic acid, neochlorogenic acid, 4-O-caffeoylquinic acid, gliosis

## Abstract

**Simple Summary:**

Neuropathic pain is defined as pain caused by a lesion or disease of the somatosensory nervous system, and it is known to have harmful effects on the quality of life of sufferers. It is characterized by hyperalgesia induced by a stimulus that normally provokes pain and allodynia due to a painless stimulus. Nowadays, no effective treatments are available to relieve neuropathic pain; hence, new pharmacological strategies are needed. Recently, it has been shown that coffee extract may prevent the development of neuropathic pain in mice subjected to spinal cord contusion, but it is unknown whether its effects are associated with the administration of the whole extract, or, in contrast, if its polyphenolic compounds could exert these effects when singly administered. Thus, the three major coffee extract polyphenols were separately administered to spinal-cord-injured mice to evaluate their pain alleviation effects compared with those exerted by whole coffee extract administration. Moreover, the reactivity of spinal cord non-neuronal cells was evaluated to elucidate whether their potential effects were associated with their modulation. The results indicated that although the major polyphenols modulated neuropathic pain development, the administration of the whole extract exerted the most beneficial effects.

**Abstract:**

It was recently shown that coffee polyphenolic extract exerts preventive effects on central neuropathic pain development, but it is unknown whether its beneficial effects are associated with only one of its major polyphenolic compounds or if the whole extract is needed to exert such effects. The main objective of this study was to determine whether the separate administration of major polyphenols from coffee extract exerts preventive effects on the development of central neuropathic pain in mice compared with the effects of the whole coffee extract. Thus, spinal-cord-injured female ICR-CD1 mice were daily treated with either coffee extract or its major polyphenolic compounds during the first week, and reflexive and nonreflexive pain responses were evaluated within the acute phase of spinal cord injury. In addition, the injury-induced gliosis and dorsal horn sprouting were evaluated with immunohistochemistry. The results showed that the coffee extract prevented spinal cord injury-induced neuropathic pain, whereas its major polyphenolic compounds resulted in reflexive pain response attenuation. Both preventive and attenuation effects were associated with gliosis and afferent fiber sprouting modulation. Overall, the results suggested that coffee extract effects may be associated with potential synergistic mechanisms exerted by its major polyphenolic compounds and not by the sole effect of only one of them.

## 1. Introduction

Spinal cord injury (SCI) is a significant medical condition that often results in severe morbidity and permanent disability. Although there is no reliable global SCI prevalence, between 40 and 80 new cases per million people per year are estimated, and between approximately 250,000 and 500,000 people are diagnosed with spinal cord injury every year [1]. Spinal cord injuries are associated with numerous complications, and among them, SCI-induced central neuropathic pain is often highlighted, since it has been shown to develop in more than half of patients [2].

Most of the available treatments for neuropathic pain do not show satisfactory results [3,4], and it remains a significant unmet medical need. In this context of a lack of effective treatments, suitable new pharmacological strategies are being studied, and among them, interest in the potential suitability of herbal medicine for pain alleviation is growing annually [5]. Although it has been suggested that herbal medicines are usually not the most potent analgesic treatments available [5], preclinical evidence shows that polyphenols derived from natural sources may exert promising pathological pain modulation effects [6]. For instance, and particularly for SCI-induced neuropathic pain, it is known that this can be successfully modulated with single polyphenols such as apocynin [7], Eugenol [8], or (-)-Epigallocatechin-3-gallate [9,10]. However, based on the current data from refractory neuropathic pain studies, it has been recently suggested that single-drug therapy can be ineffective [4]. Thus, in applying this knowledge in the field of polyphenolic treatments for pathological pain relief, a suitable strategy would be to use whole natural extracts, containing several polyphenols, which could exert synergistic effects, unlike single compounds. Per this hypothesis, our research group recently showed that natural coffee polyphenolic extract exerts preventive effects on the development of central neuropathic pain in wild-type CD-1 Swiss female mice in the acute phase of SCI. The effects of the repeated administration of coffee extract were associated with the modulation of central nervous system gliosis as well as the downregulation of chemokines and their receptors, which are known to be related to the development of pathological pain [11].

Botanical therapies containing mixtures of natural products have long attracted interest, largely owing to the potential synergistic therapeutic effects of components within the mixture [12,13], but reproducibility and potential non-controlled adverse effects are concerns that must be addressed before they can be used as reliable therapies [5,13,14]. This is not the case for coffee extract, since a commercial powdered decaffeinated variety was used, with physiological serum as the extractor to be compatible with in vivo administration. In addition, the polyphenolic content was fully characterized by HPLC, and its repeated administration in mice has shown neither hepatoxicity nor nephrotoxicity [11]. However, while it is known that coffee extract consists of a mixture of several polyphenolic molecules, it is still unknown whether its beneficial effects on the development of central neuropathic pain are associated with the potential synergistic mechanisms of the polyphenolic content of the whole extract or, in contrast, with one of the major polyphenols within the solution. Considering that polyphenols may exert several beneficial effects via different molecular pathway modulations [6], it is not unreasonable to suggest that coffee extract may exert its antinociceptive effects using synergistic mechanisms; however, not enough data are available about major coffee extract polyphenolic compounds in the prevention of SCI-induced neuropathic pain development. Hence, considering that the major polyphenolic compounds present in the coffee extract are chlorogenic acid (CGA), neochlorogenic acid, and 4-O-caffeoylquinic acid, the main objective of this work was to determine whether these polyphenols, when administered separately and in the concentration present in the coffee extract, exert preventive effects on the development of SCI-induced central neuropathic pain in mice, as compared with the effect of whole coffee extract. Moreover, the modulation of reflexive and nonreflexive pain responses, spinal cord gliosis, and afferent fiber sprouting in the dorsal horn were evaluated after treatments during the acute phase of spinal cord injury.

## 2. Materials and Methods

### 2.1. Experimental Design

This study was designed to elucidate whether the major polyphenolic compounds in coffee extract exert preventive effects in central neuropathic pain development when they are administered separately, within the acute phase of SCI. To this end, female Swiss mice subjected to mild spinal cord contusion were administered (i.p.) CGA, neochlorogenic acid, 4-O-caffeoylquinic acid, coffee extract, or saline vehicle daily during the first week after injury. These polyphenols were chosen since they are the major ones present in natural coffee extract according to high-performance liquid chromatography high-resolution mass spectrometry (HPLC-HRMS) results [11]. Reflexive pain responses and locomotor activity were evaluated weekly up to 14 days post-injury. Moreover, at the end of the experimental period, depression-like behavior was also evaluated as a nonreflexive pain response. The animals were then sacrificed to obtain spinal cord and serum samples for subsequent immunohistochemical and toxicological studies. Note that Sham-operated animals were involved in the experimental design as a control group for central neuropathic pain development. All functional and pharmacological procedures were performed in the morning and carried out as explained below.

### 2.2. Animals and Ethical Regulations

All experiments were performed with adult female Swiss CD1 mice purchased from Janvier (Le-Genest-Saint-Isle, France). In vivo experimental procedures were performed in animal facilities (Bellvitge Campus, University of Barcelona, Barcelona, Spain). Mice were acclimatized for one week before experimentation and housed 4 to 5 per cage in standard Macrolon cages (28 cm × 28 cm × 15 cm) with a wood shaving bedding. They were maintained on a 12:12 h L:D cycle at 22 ± 2 °C and given standard diet pellets ad libitum. All experimental procedures and animal husbandry were conducted following the ARRIVE 2.0 guidelines and performed according to the ethical principles of the IASP for the evaluation of pain in conscious animals [15] and the European Parliament and the Council Directive of 22 September 2010 (2010/63/EU), along with the approval of the Ethical Committee on Animal Experimentation (CEEA) of the University of Barcelona and the Department of Agriculture, Livestock, Fisheries, Food, and Natural Environment of the *Generalitat de Catalunya*, Government of Catalonia (DAAM number 9918-P3).

### 2.3. Surgical Procedure and Spinal Cord Contusion

Mice were anesthetized with sodium pentobarbital (50 mg/kg, i.p.) and placed prone on a heating pad to preserve body temperature. After the back was shaved and disinfected with povidone iodide, dorsal laminectomy was performed to expose T8–T9 thoracic spinal cord segments. To induce mild spinal cord injury, a weight drop apparatus was used (2 g; 25 mm high) [10,11,16,17]. This spinal cord contusion is known to cause spinal cord injury but preserves the ventrolateral funicles bilaterally, which is where the ascending pain pathways project [11]. After closing the wound, the animals received (i.p.) 0.5 mL saline solution to restore an eventual volemic deficit. Finally, they were allowed to recover in warmed cages with food and water ad libitum. In Sham animals, the spinal cord was exposed following the same chirurgical procedures, but it was not contused.

### 2.4. Polyphenolic Treatments

To evaluate the modulation effects of the coffee extract’s major polyphenolic compounds, thirty minutes after contusion and daily during the first week post-surgery, intraperitoneal (i.p.) administrations of the following treatments were applied: saline solution (SCI + vehicle; control group), CGA (00500590-25MG, Sigma-Aldrich, Madrid, Spain), neochlorogenic acid (94419-10MG, Sigma-Aldrich, Madrid, Spain), 4-O-caffeoylquinic acid (65969-10MG, Sigma-Aldrich, Madrid, Spain), and natural coffee extract (*n* = 5–6 mice per experimental group).

Coffee10 experimental group animals received 10 mg/kg of well-quantified and -characterized coffee extract, which has recently been shown to exert significant attenuative effects on spinal-cord-injury-induced neuropathic pain development in mice [11]. Briefly, the coffee extract was obtained from commercial decaffeinated ground roasted (dark medium) coffee (pure *Coffea canephora* and pure *Coffea arabica*). Three grams of coffee particles were mixed with 50 mL of saline solution (0.9% Vitulia Physiological Serum, Barcelona, Spain), and the solution was refluxed and stirred at 100 °C for 2 h. The solution was then filtered with chromatographic filters (Scharlau Nylon Syringe filter, ø 13 mm, 0.45 μm, NY13045200) and further filtered and sterilized with 0.22 μm vacuum filter bottles (FPE-204-250, Biofil) to avoid the presence of bacteria in the final solution. The total concentration of polyphenols in the coffee extract was calculated using the Folin–Ciocalteu assay [11,18], resulting in 2456 mg GAE/L. Further characterization using HPLC analysis revealed that CGA, neochlorogenic acid, and 4-O-caffeoylquinic acid were the major polyphenols in this extract [11]. Considering the concentration of the major polyphenols present in the characterized coffee extract, and considering the dose of 10 mg/kg to be the most effective [11], the doses of these major polyphenols administered to the mice were 1.71 mg/kg for CGA, 1.55 mg/kg for neochlorogenic acid, and 1.55 mg/kg for 4-O-caffeoylquinic acid. All polyphenols were dissolved in saline solution at concentrations of 200 mg/L.

### 2.5. Functional Evaluation

All functional tests were performed at 0 (before surgery), 7, and 14 days post-surgery, except the forced swim test, which was only performed on the 14th day post-surgery to avoid habitation that could mask nonreflexive pain response evaluation.

#### 2.5.1. Locomotor Activity

Locomotor activity was individually evaluated with the Basso Mice Scale (BMS) test [19], which ranges from 0 (hind-limb paralysis) to 9 (no locomotor disturbances). Each mouse was allowed to move freely inside an open field (72 cm × 72 cm). Testing sessions were conducted for 5 min by two trained investigators, and the final score of each animal was the mean value of both examiners.

#### 2.5.2. Reflexive Pain Response Assessment: Thermal Hyperalgesia and Mechanical Allodynia

The reflexive pain responses (thermal hyperalgesia and mechanical allodynia) were assessed according to procedures explained elsewhere [11,16,17]. An algesimeter (#37370; Ugo Basile, Comerio, Italy) was used to perform the Hargreaves method [20] for evaluating thermal hyperalgesia. Mice were allowed to acclimate for 1 h in the algesimeter. The radiant heat source was positioned under the hind paw with a time limit of 25 s to avoid skin damage when the withdrawal response was absent. Mechanical allodynia was assessed by measuring 50% withdrawal thresholds of the hind paw to von Frey monofilaments stimulation (bending force range, 0.04–2 g), following the up-down paradigm [11,17]. Mice were placed in individual compartment enclosures on a framed metal mesh floor. After 1 h of acclimatization, von Frey monofilaments were applied to the plantar surface for 2 s, with interstimulus intervals of 5–10 s. Clear paw withdrawal, shaking, or licking were considered to be a response. Both paws were evaluated and the mechanical threshold that produced 50% of responses was calculated using the Dixon formula [21]: 50% paw withdrawal threshold (g) = [(10(Xf + κδ)/10,000)], where Xf is the value (in logarithmic units) of the final von Frey filament used, κ is a fixed tabular value for the pattern of positive/negative responses, and δ is the mean difference (in logarithmic units) between stimuli.

#### 2.5.3. Nonreflexive Pain Response Assessment: Depression-Like Behavior

This response was assessed by the forced swim test. Animals were individually placed in 40 cm high × 15 cm diameter glass open cylinders containing 30 cm of water at 25 ± 1 °C, and they were forced to swim for 6 min. Their behavior was recorded (Sony HDR-CX190) to measure the mobility and immobility time of each animal. The immobility time was determined when no additional activity was observed other than the movements required to keep the head above water. A behavior is considered depressive when the animal exhibits longer immobility time when compared with the control animals’ evaluation [22,23].

### 2.6. Tissue and Serum Samples Collection

Once all in vivo functional studies were performed (14 days post-injury), the animals were carefully anesthetized with sodium pentobarbital (90–100 mg/kg; i.p.) before collecting blood and spinal cord samples. Blood samples were collected intracardially when foot reflex was absent, and breathing was located in the abdominal region. The samples were then centrifuged (15 min, 4.000 r.m.p.) and the obtained serum was quickly frozen (−80 °C) until analysis. To collect the spinal cord, it was exposed with a dorsal laminectomy, and the segment distal to T10 was extracted and immersed in fixing solution (0.3% picric acid and 4% paraformaldehyde in saline phosphate buffer PBS 0.1 M pH = 7.4) [24] for at least 15 days at 4 °C. The fixing medium was then replaced with a cryo-preservative (30% sucrose in PBS 0.1 M pH = 7.4). Samples were kept at 4 °C for at least 15 additional days.

### 2.7. Immunohistochemical Staining and Image Analysis

Spinal cord segments of 2–3 mm in length were dissected from each animal and embedded into Tissue Freezing Media (0201-08926, Leica, Barcelona, Spain). Cryostat (CM1520, Leica, Barcelona, Catalonia, Spain) sections (60 μm thick; 6–8 sections) were placed in 6-inch porcelain plates and firstly washed with phosphate-buffered saline (PBS, 0.1 M, pH = 7.4) and 0.3% Triton-X-100 in phosphate-buffered saline (PBS-Triton) and for one hour with 1% fetal bovine serum in PBS-Triton (PBS-Triton-FCS). Sections were then incubated with primary antibodies for 48 h at 4 °C in a humid chamber. Rabbit anti-glial fibrillar acid protein (GFAP, 1: 200; ab7260; ABCAM, Cambridge, UK), rabbit anti-ionized calcium-binding adapter molecule type 1 (Iba1; 1: 200; 019-19741; WAKO, Richmond, VA, USA), goat anti-calcitonin gene-related peptide (CGRP; 1:200; ab36001, ABCAM, UK), and biotinylated Bandeiraea simplicifolia Isolectin (IB4; 1:200; L2140, Merck-Sigma-Aldrich, Darmstadt, Germany) were used to evaluate astrocytes, microglia cells, peptidergic, and non-peptidergic C-type afferent pain fibers, respectively. Then, sections were washed in PBS-Triton and incubated with Cy3-conjugated donkey anti-rabbit or anti-goat secondary antibody (1:200; Jackson ImmunoResearch, West Grove, PA, USA), whereas IB4-immunostained sections were incubated with Cy3-conjugated avidin (Jackson ImmunoResearch) for 24 h at 4 °C in a humid chamber. The immunostained sections were mounted on pregelatinized slides following a wash with PBS-Triton and PBS, dehydrated in ethanol baths in increasing concentrations (70%, 96%, 100%), and cover-slipped with DPX mounting media (1.01979.0500, Merck, Germany).

Dorsal horn images of immunolabeled sections were taken (×200) with a digital camera (FMVU-13S2C-CS; Point Gray Research, Richmond, BC, Canada) coupled to an epifluorescence microscope (Leica DMRXA; Leica Microsystems, Barcelona, Spain). Images were analyzed using the Image J software (Image Processing and Analysis in Java, National Institute of Health, NIH, v. 1.52a, USA) to evaluate the degree of astrogliosis determined by the GFAP immunoreactive area. The imaging of the sections immunolabeled for IBA1 was performed for reactive and nonreactive microglial cells and was expressed as a percentage of two phenotypes. This percentage was considered an index of the degree of microgliosis. Furthermore, spinal cord images (×100) of CGRP- and IB4-immunostained sections were analyzed by measuring their respective reactivity areas on the dorsal horn. The immunostaining area for GFAP, CGRP, and IB4 was related to the area of interest and expressed as the mean of the relative area (%) ± SEM.

### 2.8. Biochemical Analysis of Hepatotoxicity and Nephrotoxicity

Serum samples were analyzed to determine the potential hepatotoxicity and nephrotoxicity processes associated with the polyphenolic treatments. The levels of alanine aminotransferase (ALT/GPT; # 11533, BioSystems, Barcelona, Spain), aspartate aminotransferase (AST/GOT; #11531, BioSystems, Barcelona, Spain), and urea-BUN (# 11536, BioSystems, Barcelona, Spain) were analyzed in the serum samples by using commercial assay kits [11].

### 2.9. Statistical Analysis

All functional and histological analyses were performed in a blinded manner using a numeric code for each mouse. The normal distribution of the data was analyzed with a Kolmogorov–Smirnov test before further applying parametric or non-parametric statistical analyses. In case of normal distribution, the repeated measures MANOVA (Wilks’ criterion) and analysis of variance (ANOVA), followed by Duncan’s test when applicable, were used for data analysis. When data did not follow a normal distribution, they were analyzed using a Friedman test for non-parametric repeated measures, followed by Bonferroni’s test. Data are illustrated in figures as mean ± SEM or median ± IQR. In all analyses, the significance level α was set at 0.05, using the IBM SPSS 25.0 statistical package for Windows (IBM Corp. Released 2017; Armonk, NY, USA).

## 3. Results

### 3.1. Major Coffee Extract Polyphenolic Compounds Significantly Attenuate Spinal-Cord-Injury-Induced Reflexive Pain Responses

**Figure 1:**
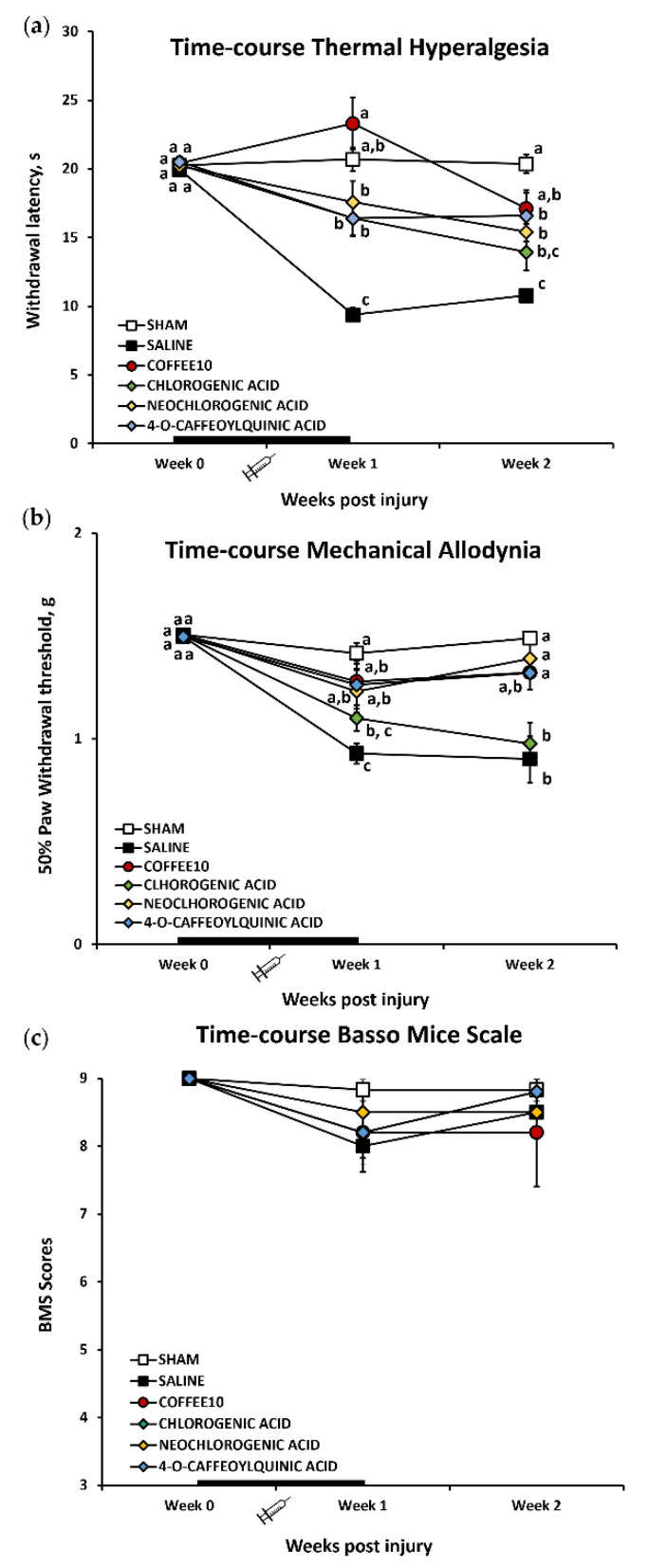
Effects of major coffee extract polyphenolic compounds on reflexive pain responses and locomotor activity: (**a**) thermal hyperalgesia; (**b**) mechanical allodynia; (**c**) BMS scores. Data are illustrated as mean ± SEM. a–c: not sharing a letter means significant differences, *p* < 0.05. Administration week (W0 to W1) is indicated with a thicker line.

The Kolmogorov–Smirnov normality test revealed a normal distribution of thermal hyperalgesia data in all the timepoints of the functional assessment (*p* > 0.05). The MANOVA analysis of thermal hyperalgesia indicated significant effects on the week (*p* < 0.001) and treatment (*p* < 0.001) factors and significant interaction for week × treatment (*p* < 0.001). Upon further ANOVA analysis, significant group differences were found in post-injury weeks 1 and 2 (*p* < 0.001). Concretely, the subsequent post hoc analysis revealed a significant (*p* < 0.005) increase in paw withdrawal latency to thermal stimulation in all polyphenolic treatment groups (CGA, neochlorogenic acid, and 4-O-caffeoylquinic acid) in comparison with the SCI-saline group (Figure 1a). Moreover, only Coffee10 mice showed a significant (*p* < 0.005) increase in paw withdrawal latency to thermal stimulation when compared with either SCI-saline or other polyphenolic treatment groups, reaching the same reflexive response level as the Sham group (Figure 1a). At the end of the experimental period (week 2), while neochlorogenic acid and 4-O-caffeoylquinic acid animals still showed a significant (*p* < 0.005) increase in paw withdrawal latency when compared with SCI-saline, chlorogenic mouse latency scores could not be completely distinguished from the SCI-saline group. Moreover, Coffee10-mice still showed a significant increase in paw withdrawal latency when compared with the SCI-saline group, similar to the Sham group scores (Figure 1a). Overall, these results indicated that thermal hyperalgesia development in SCI-saline mice was significantly prevented by the Coffee10 treatment up to 2 weeks after injury, whereas CGA, neochlorogenic acid, and 4-O-caffeoylquinic acid exerted significant attenuation effects during the same experimentation period.

In contrast to thermal hyperalgesia, the Kolmogorov–Smirnov normality test revealed that the mechanical allodynia data did not follow a normal distribution at any timepoint in the experimental period (*p* < 0.013). The Friedman test revealed that the distribution of the mechanical allodynia data significantly varied during the experimental period (*p* < 0.001) and the Kruskal–Wallis test indicated differences between groups at weeks 1 (*p* = 0.06) and 2 (*p* = 0.04) post-injury. A subsequent post hoc test revealed a significant mechanical sensitivity increase in the SCI-saline group when compared with the Sham animals during the whole experimental period (*p* = 0.02) (Figure 1b). In contrast, no differences were recorded between Sham and Coffee10, neochlorogenic acid, or 4-O-caffeoylquinic acid, neither in week 1 (*p* > 0.171) nor in week 2 (*p* > 0.329) post-injury (Figure 1b). In parallel, the latter treatment groups showed a significant decrease in mechanical allodynia when compared with SCI during the whole experimental period (*p* > 0.05). Ultimately, while CGA showed decreased mechanical sensitivity similar to the levels of the other polyphenols at week 1 post-injury (*p* > 0.05), no significant differences were recorded at week 1 (*p* = 0.126) or week 2 (*p* = 0.792) when compared with SCI animals (Figure 1b). Overall, these findings indicate that mechanical allodynia development in SCI-saline mice was significantly prevented by Coffee10, neochlorogenic acid, and 4-O-caffeoylquinic acid treatments up to 2 weeks after injury since these treated animals reached the same reflexive response level as the Sham group. In contrast, CGA exerted significant attenuation effects only up to one week after injury.

In parallel to the reflexive pain response assessment, locomotor activity was also evaluated, and we obtained BMS scores higher than seven (Figure 1c). The Kolmogorov–Smirnov normality test revealed that the data did not follow a normal distribution (*p* <0.0001), but while the Friedman test revealed that the distribution of BMS scores varied significantly over time (*p* < 0.001), the post hoc indicated no differences between the groups at any timepoint (Figure 1c). Thus, all animals showed similar recuperation after surgery during the experimental period, and none of the groups showed important locomotor disturbances that could interfere with reflexive pain outcomes.

### 3.2. Major Coffee Extract Polyphenolic Compounds Prevent the Development of Depression-Like Behavior after Spinal Cord Contusion

**Figure 2:**
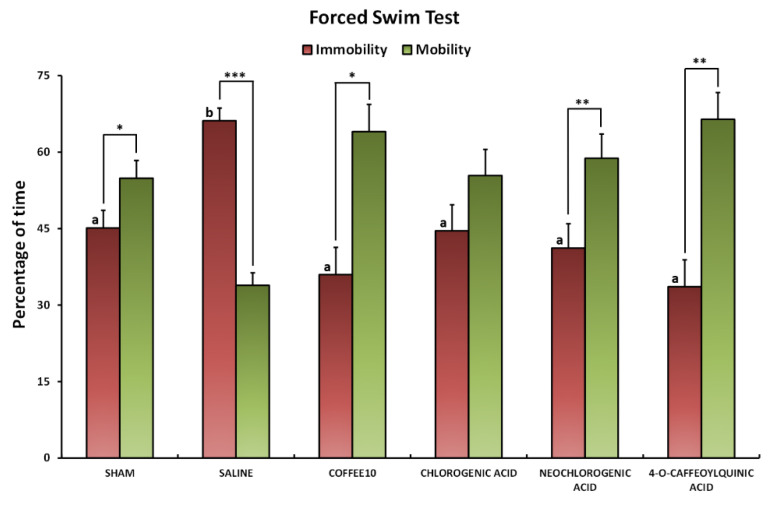
Effects of major coffee extract polyphenolic compounds on depression-like behavior fourteen days post-injury. Data are illustrated as mean ± SEM. a,b: not sharing a letter means significant differences in %immobility time, *p* < 0.05; intra-groups significant differences: *** *p* < 0.0001, ** *p* < 0.001, * *p* < 0.05 %immobility vs. %mobility.

The Kolmogorov–Smirnov normality test revealed a normal distribution of forced swim data at 2 weeks post-injury (*p* = 0.200). The ANOVA analyses showed significant group differences in the percentage of immobility in the Porsolt test (*p* < 0.001). The subsequent post hoc analysis revealed no significant immobility time in any of the treated groups when compared with the Sham animals (*p* > 0.05), and all of them showed a significantly decreased immobility time in comparison with the SCI-group (*p* < 0.05) (Figure 2). In parallel, while the intra-group statistical analysis of %mobility versus %immobility showed significantly higher mobility versus mobility in the Sham (*p* = 0.029), Coffee10 (*p* = 0.006), neochlorogenic (*p* = 0.039), and 4-O-caffeoylquinic (*p* = 0.02) animals, significantly higher immobility was found in SCI-saline animals (*p* < 0.0001) (Figure 2). In contrast, a lack of significant differences between immobility and mobility were shown in CGA-treated animals (*p* = 0.171) (Figure 2). In summary, these results indicate that the percentage of immobility increased in SCI-saline mice at 2 weeks post-injury and that it was significantly prevented by Coffee10, neochlorogenic acid, 4-O-caffeoylquinic acid, and CGA treatments, although animals administrated with the latter showed similar levels of mobility and immobility.

### 3.3. Major Coffee Extract Polyphenolic Compounds Significantly Attenuate Spinal Cord Gliosis, but the Whole Coffee Extract Exerts the Most Effective Effect

**Figure 3:**
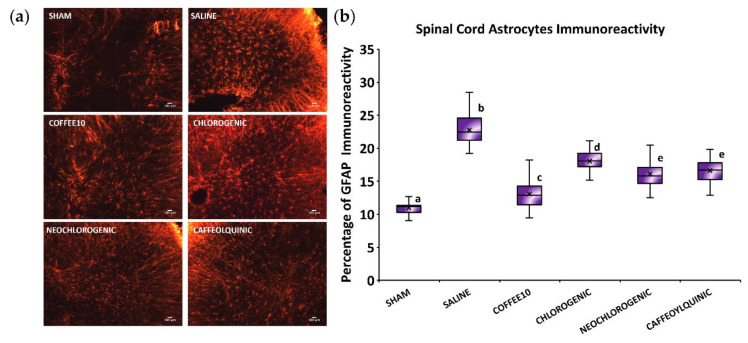
Spinal cord astroglia after polyphenolic treatments fourteen days post-injury: (**a**) Representative GFAP images of dorsal horn for each group. Scale bars = 100 μm; (**b**) Percentage of GFAP immunoreactivity. Data are illustrated as median ± IQR, and the x indicate the mean. a–d: not sharing a letter means significant differences, *p* < 0.05.

The Kolmogorov–Smirnov normality test revealed a normal distribution of GFAP-immunoreactivity data at 2 weeks post-injury (*p* = 0.200), and the ANOVA analyses showed significant group differences (*p* < 0.001). A post hoc analysis revealed a significant decrease in astrogliosis in all treated groups when compared with the SCI-saline group (*p* < 0.05) (Figure 3). Furthermore, although neochlorogenic acid, 4-O-caffeoylquinic acid, and chlorogenic acid modulated astrogliosis, the analysis revealed that Coffee10 was the most effective in preventing astrogliosis.

**Figure 4:**
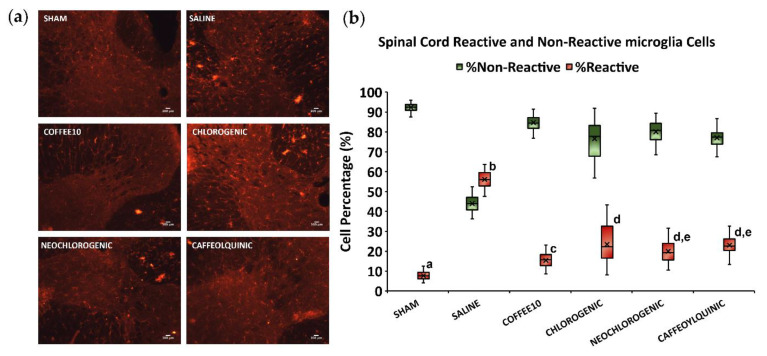
Spinal cord microglia reactivity after polyphenolic treatments fourteen days post-injury: (**a**) Representative IBA1 images of dorsal horn for each group. Scale bars = 100 μm; (**b**) Percentage of reactive and non-reactive microglia cells. Data are illustrated as median ± IQR, and the x indicate the mean. a–e: not sharing a letter means significant differences, *p* < 0.05.

As for microgliosis, the Kolmogorov–Smirnov normality test revealed that the data did not follow a normal distribution (*p* < 0.0001). The Kruskal–Wallis test indicated significant differences between groups (*p* < 0.0001) in both the percentage of reactive and non-reactive microglia cells. A subsequent post hoc test showed a significant (*p* < 0.0001) increase in the percentage of reactive microglia cells in SCI-saline mice when compared with Sham animals. In parallel, all treatment groups showed a significant decrease in the reactive cell percentage in comparison with the SCI-saline group (*p* < 0.0001), but none of them reached the Sham group’s level of percentage since they all significantly differed from this control group (*p* < 0.0001) (Figure 4). Nevertheless, apart from the Sham mice, Coffee10 animals showed the lowest significant percentage of reactive microglia cells when compared with the rest of the treatment groups, whereas chlorogenic-acid-treated animals showed the highest. Overall, all treatments decreased the percentage of reactive microglia cells observed in the spinal cords of SCI-saline mice, but Coffee10 exerted the most effective effect, whereas chlorogenic acid exerted the lowest when treatments were compared.

### 3.4. Major Coffee Extract Polyphenolic Compounds Significantly Attenuate Spinal-Cord-Injury-Induced Sprouting of Afferent Fibers in the Dorsal Horn, but the Whole Coffee Extract Exerts the Most Effective Effect

**Figure 5:**
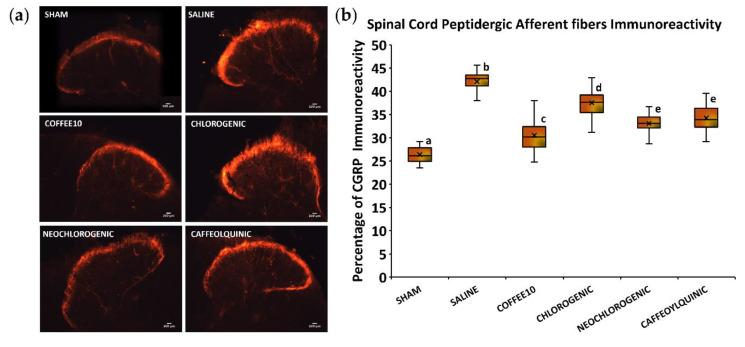
Sprouting of peptidergic afferent nerve fibers in the spinal cord after polyphenolic treatments fourteen days post-injury: (**a**) Representative CGRP images for each group. Scale bars = 100 μm. (**b**) Percentage of dorsal horn CGRP-immunoreactivity. Data are illustrated as median ± IQR, and the x indicate the mean. a–e: not sharing a letter means significant differences, *p* < 0.05.

As for spinal cord peptidergic afferent fibers, the Kolmogorov–Smirnov normality test revealed a normal distribution of CGRP immunoreactivity data at 2 weeks post-injury (*p* = 0.200), and the ANOVA analyses showed significant group differences (*p* < 0.001). A post hoc analysis revealed a significant decrease in CGRP immunoreactivity in all treated groups when compared with SCI-saline (*p* < 0.05) (Figure 5). Furthermore, it revealed that Coffee10 showed the most effective sprouting prevention, and then this modulation was relatively lower, in a graded manner, after neochlorogenic acid, 4-O-caffeoylquinic acid, and chlorogenic acid treatments, respectively.

**Figure 6:**
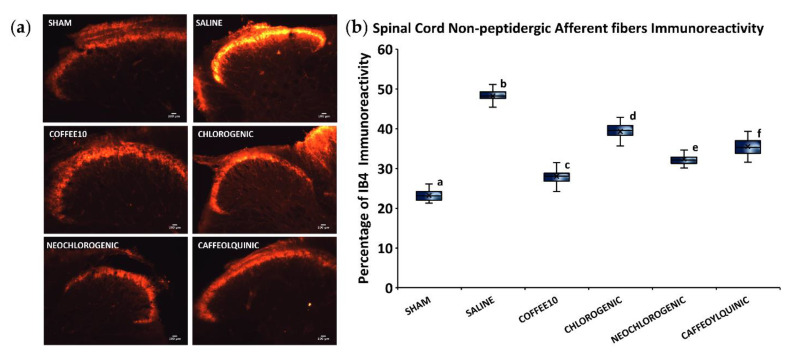
Sprouting of non-peptidergic afferent nerve fibers in the spinal cord after polyphenolic treatments fourteen days post-injury. (**a**) Representative IB4 images for each group. Scale bars = 100 μm. (**b**) Percentage of dorsal horn IB4 immunoreactivity. Data are illustrated as median ± IQR, and the x indicate the mean. a–f: not sharing a letter means significant differences, *p* < 0.05.

Regarding non-peptidergic afferent fibers, the Kolmogorov–Smirnov normality test revealed a normal distribution of IB4-immunoreactivity data at 2 weeks post-injury (*p* = 0.200), and the ANOVA analyses showed significant group differences (*p* < 0.001). A post hoc analysis revealed a significant decrease in IB4 immunoreactivity in all treated groups when compared with the SCI-saline group (*p* < 0.05) (Figure 6). Furthermore, similar to the peptidergic immunostaining assessment, we found that Coffee10 most effectively prevented sprouting and then, this modulation was relatively lower in a graded manner, after neochlorogenic acid, 4-O-caffeoylquinic acid, and chlorogenic acid treatments, respectively.

### 3.5. No Signs of Systemic Toxicity, Hepatotoxicity, or Nephrotoxicity Were Associated with the Polyphenolic Treatments

**Figure 7:**
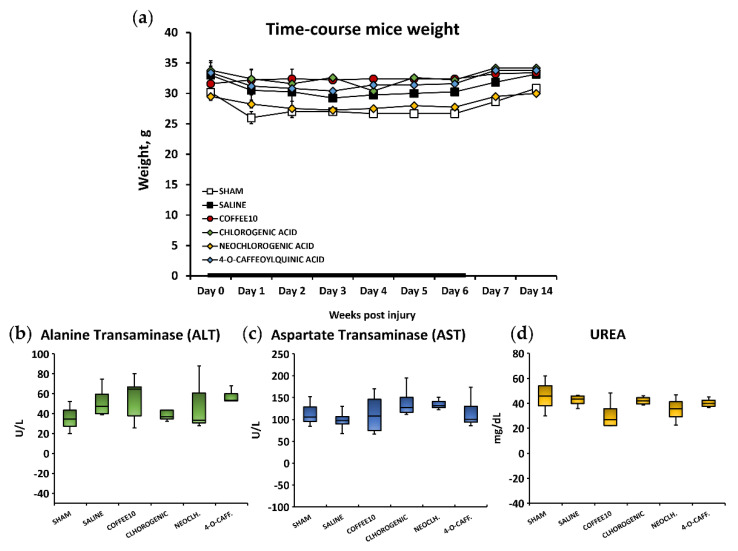
(**a**) Time−course weight. Data are illustrated as mean ± SEM. Administration week (W0 to W1) is highlighted with a thicker line. No differences between groups are shown over time. Serum biomarkers quantification of (**b**,**c**) hepatotoxicity and (**d**) nephrotoxicity of each experimental group fourteen days post-injury. Data are illustrated as median ± IQR, and the x indicate the mean. No differences between groups are shown in any of the parameters.

Following animal welfare protocol supervision based on the guidelines of Morton, D.B and Griffiths, P.H. [25], changes in coat and skin, the vibrissae of the nose, and nasal secretions, as well as signs of autotomy in the hind paw and/or forepaw and aggressiveness were not detected in the mice after either spinal cord contusion or Sham surgery, nor after treatment administrations at any time during the experimental period. Regarding weight control, the Kolmogorov–Smirnov normality test revealed that the data did not follow a normal distribution (*p* < 0.0001). Then, while the Friedman test revealed that the distribution of weight data varied significantly over time (*p* < 0.001), the Kruskal–Wallis test indicated no significant differences between the groups at any timepoint (*p* < 0.051) (Figure 7a). These results indicated that neither surgeries nor treatments had significant effects on the animals’ weights.

As for the biochemical analysis of hepatotoxicity and nephrotoxicity, the Kolmogorov–Smirnov normality test revealed a normal distribution of the ALT, AST, and UREA data (*p* > 0.05). A further ANOVA analysis of the ALT, AST, and UREA biomarkers of the mice serum revealed non-significant group differences (*p* > 0.353) (Figure 7b,c). Altogether, these findings suggest that there would be no systemic toxicity associated with the administration of polyphenols and no hepatotoxic or nephrotoxic effects.

## 4. Discussion

Inflammogenesis of secondary SCI plays a major role in the central pain sensitization phenomena associated with neuropathic pain development [26]. In this pathophysiological process, activated spinal cord glial cells releasing pro-inflammatory mediators are key cellular contributors in the development of abnormal physiological conditions, which result in maladaptive synaptic circuits and contribute to the development of pathological pain [27]. Accordingly, new pharmacological strategies targeting glial modulation may be potentially suitable to manage SCI-induced central neuropathic pain. In this context, the results from our research group showed that coffee extract (Coffee10), consisting of several polyphenolic compounds characterized by HPLC, reduced the development of SCI-induced neuropathic pain by modulating spinal cord glia in the mouse model [11]. However, lesser was known about the effect of its major polyphenolic compounds on the development of SCI-induced neuropathic pain when they were singly administrated. As a result, the repeated administration of the Coffee10 extract better reduced SCI-induced neuropathic pain within the acute phase compared with separate treatments using the main polyphenolic compounds contained in the extract. Therefore, combining the main effects of the polyphenols may be potentially associated with the beneficial effects of the complete extract. In other words, our findings suggested that the analgesic effects of Coffee10 extract would be associated with potentially synergistic mechanisms exerted by the main polyphenolic compounds. Nevertheless, considering the lack of information about the effects of polyphenols on SCI-induced neuropathic pain, the attenuation effects detected in the present work may also yield an interesting result, increasing the chances of a new pharmacological design to treat central neuropathic pain. Before this work, little information was available about the modulation effects of these polyphenols on SCI-induced neuropathic pain.

As for chlorogenic acid (3-caffeoylquinic acid), which is the main polyphenolic compound found in the coffee extract, it is a cinnamate ester obtained by formal condensingation of carboxy group of trans-caffeic acid with the 3-hydroxy group of quinic acid [28]. While antihyperalgesic properties have been reported after its administration in animal models of pathological pain [29,30,31,32], no results have shown its effects on SCI-induced neuropathic pain. However, CGA is also known to exert anti-inflammatory effects [29,32], and considering that spinal cord contusion leads to an inflammatory response, CGA present in the coffee extract may contribute to the attenuation of neuropathic pain development because of its anti-inflammatory properties. These effects may be related to its significant attenuative effects on glia modulation, leading to the reduced release of pro-inflammatory cytokines, which are known to be upregulated in central pathological pain status [26]. It has been reported that CGA may modulate chronic-constriction-injury-induced mechanical allodynia in rats [29], and its antinociceptive effects may be associated with a reduction in oxidative and inflammatory mediator activity [29,33,34], closely related to the induction of neuropathic pain processes. Neochlorogenic acid (5-O-caffeoylquinic acid) is an isomer of CGA and is the second-most concentrated polyphenolic compound found in the coffee extract. It is a cinnamate ester obtained by formal condensation of the carboxy group of trans-caffeic acid with the 5-hydroxy group of quinic acid [35]. While no information has been reported about neochlorogenic antinociceptive effects on SCI-induced neuropathic pain, this isomer has been found to also be an antioxidant and to have anti-inflammatory properties [36], indicating its potential effects on the modulation of SCI pathophysiology. Neochlorogenic acid has also been suggested to reduce extracellular signal-regulated kinases (ERK), Jun-amino-terminal kinase (JNK), MAPK phosphorylation, and NF-κB activation [36,37], which are intracellular signaling cascades that play pivotal roles in the development of neuropathic pain [16,38]. Finally, 4-O-caffeoylquinic acid, the third major coffee extract polyphenolic compound, is a cinnamate ester obtained by formal condensation of the carboxy group of trans-caffeic acid with the 4-hydroxy group of (+)-quinic acid [39]. This is the least studied polyphenol of all those mentioned since only antioxidant properties have been associated with its administration [40], and no pharmacological effects, either on pathological or specific neuropathic pain, have been described yet. Hence, for the first time, the present work describes the antinociceptive effects of this polyphenol on SCI-induced neuropathic pain associated with the glial modulation of the spinal cord.

Other available information that may be interesting to consider is that CGA reduces the activation of BV2 microglia [41,42] and astroglia reactivity [43] in vitro after LPS stimulation, and neochlorogenic acid also reduces BV2 microglia reactivity after LPS stimulation [44]. In the context of tumor cells, it has been observed that CGA can bind to EGFR and inhibit various intracellular cascades, leading to the suppression of cell growth and proliferation [45,46]. In the central nervous system, reactive glial cells overexpress EGFR [47]. It is well known that EGFR is implicated in the migration and proliferation of reactive microglia cells [48], and EGFR-MAPK inhibition reduces microglia activation and cytokine production after SCI [49]. Taken together, all these findings suggest that CGA could reduce glial reactivity and the production of proinflammatory mediators by binding to EGFR, as well as the inhibition of intracellular MAPK-associated cascades. Moreover, CGA also blocks Toll-like receptors (e.g., TLR2, TRL4, TLR9) in reactive BV2 microglia, reducing the release of pro-inflammatory cytokines [42,50]. In addition, experimental evidence suggests that CGA inhibits NLRP3 inflammasome activation [51,52] and mediates IL-1β release, contributing to central sensitization and hyperalgesia [53,54]. Chemical studies have demonstrated that CGA can bind to beta-2-adrenergic receptors [55]. Beta-2-adrenergic receptors are involved in the reactivation of microglia cells, so the application of adrenergic agonists induces microglial reactivity, while the antagonists reduce this microglial reactivity [56,57,58]. This evidence suggests that there are several molecular mechanisms involved in the modulation of glial reactivity caused by CGA treatment, reducing pro-inflammatory mediators and hyperalgesia.

Besides the results discussed so far, all three major coffee extract polyphenols evaluated, as well as the whole coffee extract Coffee10, attenuated both peptidergic and non-peptidergic afferent fibers sprouting in the dorsal horn, which were significantly increased in spinal-cord-injured mice. These phenomena may also contribute to the modulation of neuropathic pain since increased density of CGRP immunopositive C-fibers in the dorsal horn laminae parallels the development of pain behavior [59,60]. In addition, the increased sprouting of isolectin B4-positive afferent fibers may also play a significant role in the development of at least SCI-induced mechanical allodynia [61,62]. These results may be associated with glial activation after SCI, as observed in the present work, since some mediators released by them would stimulate afferent fiber sprouting. For instance, it has been reported that peptidergic and non-peptidergic nerve fibers are sensible to NGF and ATP mediators [63], respectively, causing their hyperexcitability, and it has been shown that these mediators can be released by activated glia cells [64]. That is, glia-activated mediators may be responsible for the hyperexcitability of afferent fibers, and they probably also induce the sprouting of these fibers in the dorsal horn after spinal cord contusions. Thus, our results suggest that polyphenolic compounds may modulate the sprouting of dorsal afferent fibers by modulating both astroglia and microglia activation.

The sprouting of nociceptive afferent fibers in the dorsal horn of the spinal cord is influenced by the levels of growth factors such as GDNF and NGF. Specific, IB4-positive nociceptive afferent fibers are sensitive to GDNF [65], whereas CGRP-positive afferent fibers are sensitive to NGF [66]. It is known that reactive glial cells secrete GDNF and NGF [67,68,69]. Likewise, it is also known that an increase in the immunoreactivity area reflects sprouting rather than peptide upregulation [70]. In this context, the changes in IB4 and CGRP immunoreactivity observed in the dorsal horn of our SCI experimental animals—treated with the different extracts compared with the control group—could be explained by the modulation of glial reactivity, to a variable degree, with the growth factor levels influencing the sprouting of nociceptive afferent fibers.

On the other hand, while the major polyphenolic compounds of the coffee extract attenuated but did not completely prevent reflexive pain responses, depression-like behavior (nonreflexive pain response) developed in SCI mice was prevented after the polyphenolic treatments. These results cannot be related to locomotor disturbances since no major alterations were found in the BMS score test. It has been reported that plastic changes can occur in supraspinal structures after SCI [11,16], and this may be associated with alterations both of pathological pain modulation and nonreflexive pain response development. It was recently shown that SCI-related supraspinal neuron-glia cross-talking can be modulated by coffee extract administration [11], indicating that polyphenols may modulate gliosis not only in the spinal cord but also in supraspinal structures. As discussed so far, it is not unreasonable to hypothesize that polyphenols may also reduce these plastic changes in the supraspinal structures associated with emotional behaviors, such as the amygdala and prefrontal cortex, preventing SCI-related mood disorders such as depression-like behavior. Although some data suggest low polyphenol availability in the brain and plasma [71,72], other in vivo studies have shown that polyphenolic compounds may penetrate the blood–brain barrier irrespective of their mode of administration [73,74,75,76,77], suggesting their potential therapeutic importance in supraspinal structures dysfunction. Nonetheless, new studies should be designed to elucidate this hypothesis. Finally, but not least, it is important that these results were determined using female mice since it is known that they show higher chronic pain prevalence and higher vulnerability to comorbid pain and development of emotional disorders [78,79].

Although not evaluated in this study, natural polyphenolic compounds have been used to reduce oxidative stress cascades, glutamate-mediated neurotoxicity cascades, neuroinflammation cascades (e.g., p38-MAPK, NF-kappa-B), and neuro-apoptosis cascades (e.g., Bax/Bcl-2, Caspases 3/9, p53) observed in the secondary phase of SCI. Polyphenolic compounds also regulate extrinsic degenerative mechanisms, such as blocking the effects of Nogo on its receptors and the Nogo signaling pathway, suppressing the formation of glial scars, and gliosis, including the release of glial mediators [80]. In summary, polyphenol treatments promote the protection of spinal cord parenchyma, reducing the formation of chemical mediators implicated in the degeneration of the spinal cord [80], as well as in the development of neuropathic pain [81].

Taking all the outcomes of the present work together, to the best of our knowledge, this is the first study showing that, although the major polyphenolic compounds of the coffee extract are potential pharmacologic strategies to modulate central neuropathic pain development after SCI without systemic toxicity, none of them exerted greater effects that the whole extract at the same concentrations. However, it is also worth mentioning that, although the results suggest that the potential synergistic activity of the different polyphenols would be associated with the pharmacological effects of the complete extract, it is still unknown whether the antinociceptive effects of the coffee extract depend on the activities of the three polyphenols or, on the contrary, if the combination of two of them exerts similar effects to those of the whole extract. While the neochlorogenic and 4-O-caffeoylquinic acids significantly attenuated reflexive pain responses up to 14 days post-injury, the CGA showed such an effect mainly up to 7 days. Thus, it may be that the combinatorial administration of the first two may be as effective as the administration of whole coffee extract. Hence, new experiments should be designed to explore this new hypothesis.

## 5. Conclusions

The major polyphenols of coffee extract administered for one week after SCI, at the concentration in which they are found in the extract, attenuate thermal hyperalgesia development in female mice. However, only the repeated administration of whole Coffee10 extract reduced the development of SCI-induced neuropathic pain during this phase to a greater degree. On the other hand, Coffee10, neochlorogenic acid, and 4-O-caffeoylquinic acid, separately administered, prevented mechanical allodynia, whereas CGA only exerted analgesic effects up to one week after injury. Regarding nonreflexive pain responses, all administrations of the coffee extract’s major polyphenolic compounds resulted in significant preventive effects on depression-like behavior, which significantly developed in SCI-saline female mice. These emotional deficits may not be attributable to locomotor disturbances according to BMS scores. Hence, these findings suggest that nonreflexive pain responses may be prevented not only by the administration of whole coffee extract but also by its major polyphenolic compounds.

As for mechanistic insights, administrations of coffee extract and its major polyphenolic compounds resulted in the significant modulation of both microgliosis and astrogliosis development in the spinal cord of SCI-saline female mice up to 14 days post-injury but Coffee10 was the treatment that showed the most significant preventive effects. Moreover, coffee extract and its major polyphenolic compounds also significantly modulated both peptidergic and non-peptidergic fiber sprouting development, with the whole coffee extract administration being the treatment that showed the most significant effects.

Taken together, all these findings suggest that the preventive effects of coffee extract may be associated with potentially synergistic mechanisms exerted by its main polyphenolic compounds, indicating that this extract and a constitutive therapy involving various polyphenols may be potential pharmacological treatments, adequate to prevent the development of spinal cord injury-induced neuropathic pain. Considering that neither the coffee extract nor any of its main polyphenolic compounds triggered systemic toxicity, such a treatment may be safe.

## Data Availability

All data generated or analyzed during this study are included in this published article.
